# Effect of Saw Palmetto Supplements on Androgen-Sensitive LNCaP Human Prostate Cancer Cell Number and Syrian Hamster Flank Organ Growth

**DOI:** 10.1155/2016/8135135

**Published:** 2016-05-04

**Authors:** Alexander B. Opoku-Acheampong, Kavitha Penugonda, Brian L. Lindshield

**Affiliations:** Department of Food, Nutrition, Dietetics and Health, Kansas State University, Manhattan, KS 66506, USA

## Abstract

Saw palmetto supplements (SPS) are commonly consumed by men with prostate cancer. We investigated whether SPS fatty acids and phytosterols concentrations determine their growth-inhibitory action in androgen-sensitive LNCaP cells and hamster flank organs. High long-chain fatty acids-low phytosterols (HLLP) SPS ≥ 750 nM with testosterone significantly increased and ≥500 nM with dihydrotestosterone significantly decreased LNCaP cell number. High long-chain fatty acids-high phytosterols (HLHP) SPS ≥ 500 nM with dihydrotestosterone and high medium-chain fatty acids-low phytosterols (HMLP) SPS ≥ 750 nM or with androgens significantly decreased LNCaP cell number (*n* = 3; *p* < 0.05). Five- to six-week-old, castrated male Syrian hamsters were randomized to control (*n* = 4), HLLP, HLHP, and HMLP SPS (*n* = 6) groups. Testosterone or dihydrotestosterone was applied topically daily for 21 days to the right flank organ; the left flank organ was treated with ethanol and served as the control. Thirty minutes later, SPS or ethanol was applied to each flank organ in treatment and control groups, respectively. SPS treatments caused a notable but nonsignificant reduction in the difference between left and right flank organ growth in testosterone-treated SPS groups compared to the control. The same level of inhibition was not seen in dihydrotestosterone-treated SPS groups (*p* < 0.05). Results may suggest that SPS inhibit 5*α*-reductase thereby preventing hamster flank organ growth.

## 1. Introduction

Prostate cancer is the most common non-skin cancer in men and is projected to account for 21% of US male cancer cases in 2016 [1]. Most prostate cancers rely on androgens for growth at the initial stages of development; thus inhibiting androgen production or blocking its action may be useful approaches in early treatment or prevention of prostate cancer [2]. 5*α*-reductase 1, 5*α*-reductase 2, and 5*α*-reductase 3 isoenzymes are potential targets because they convert testosterone to the more potent dihydrotestosterone (DHT), which binds with up to 10-fold higher affinity to the androgen receptor than testosterone [3, 4] to stimulate prostate cancer growth.

Saw palmetto extracts inhibited 5*α*-reductase and decreased growth of human prostatic cells* in vitro* [5–7], decreased prostate tumor progression and prostate DHT concentrations in transgenic adenocarcinoma of the mouse prostate (TRAMP) mice [8], decreased prostate growth and hyperplasia in castrated, DHT-implanted, sulpiride-treated rats [9], inhibited testosterone-induced prostate growth [10] and hyperplasia [11] in rats, and decreased prostate specific antigen (PSA) levels in men with enlarged prostates [12].

The antiandrogenic action of saw palmetto supplements (SPS) has been attributed to their fatty acid and phytosterol content. Most SPS are rich sources of the medium-chain saturated fatty acids (FA) laurate and myristate [13]. Multiple studies [14–19] suggest that SPS fatty acids are responsible for their ability to inhibit 5*α*-reductase enzymes. However, the specific fatty acid(s) purported to be responsible for this inhibition differs between publications. For example, *γ*-linolenic acid inhibited testosterone-treated but not DHT-treated growth of androgen-sensitive hamster flank organs [17]. Oleate and laurate inhibited 5*α*-reductase activity in rat liver [18], and laurate and myristate inhibited epithelial and stromal 5*α*-reductase activity in human benign prostatic hyperplasia (BPH) [19]. There are also multiple studies that suggest that SPS phytosterols (campesterol, stigmasterol, and *β*-sitosterol) inhibited 5*α*-reductase in hamster prostate [20] and decreased human prostate cancer cell/tumor growth [21–23] and BPH symptoms in men [24].

There is growing evidence to suggest that single-agent interventions identified using a reductionist approach are not an effective strategy for decreasing cancer risk [25]. Rather than taking a reductionist approach to try to identify the bioactive compound(s) in SPS, we set out to determine the efficacy of SPS with different fatty acid and phytosterol profiles (high long-chain FA-low phytosterols (HLLP), high long-chain FA-high phytosterols (HLHP), and high medium-chain FA-low phytosterols (HMLP)) in decreasing androgen-sensitive LNCaP human prostate cancer cell number and androgen-sensitive Syrian hamster flank organ growth.

The cell culture studies determined whether SPS decrease LNCaP human prostate cancer cell number without inducing cytotoxicity with and without androgen treatment. The Syrian hamster was selected for further studies because its flank organs have dermal melanocytes, sebaceous glands, and hair follicles that are highly dependent on androgens for development [26, 27]. SPS were applied to castrated, male Syrian hamster flank organs treated with testosterone or DHT to determine whether SPS impact androgen-treated flank organ growth as a measure of antiandrogenic action. We hypothesized that HMLP SPS would significantly decrease LNCaP cell number and Syrian hamster flank organ growth compared to HLLP and HLHP SPS.

## 2. Materials and Methods

### 2.1. Saw Palmetto Supplements Fatty Acids and Phytosterols Extraction and Quantification

Saw palmetto supplements' (GNC Herbal Plus SPS, GNC Corporation, Pittsburgh, PA; Jarrow Formulas SPS, Superior Nutrition and Formulation, Los Angeles, CA; Doctor's Best SPS, All Star Health, Huntington Beach, CA) fatty acid and phytosterol profiles were analyzed according to previously described methods [28] and categorized into high long-chain FA-low phytosterols (HLLP), high long-chain FA-high phytosterols (HLHP), and high medium-chain FA-low phytosterols (HMLP) SPS groups, respectively.

### 2.2. Cell Culture and Reagents

LNCaP cells (androgen-dependent, prostate adenocarcinoma cells derived from lymph node metastasis (CRL-1740), American Type Culture Collection, Manassas, VA) were grown in Roswell Park Memorial Institute- (RPMI-) 1640 medium (GIBCO Invitrogen, Carlsbad, CA) containing 2 g/L glucose supplemented with 10% fetal bovine serum (Atlanta Biologicals, Inc., Flowery Branch, GA) at 37°C in a 5% CO_2_, 95% air-humidified atmosphere incubator. LNCaP cells were maintained in T-75 tissue culture flasks (TPP, Midwest Scientific, Inc., Valley Park, MO) with media changed every 72 hours.

### 2.3. LNCaP Cell Treatment

LNCaP cells (passage number ≤ 18) were plated at a density of 20,000 cells per well in 96-well plates (Fisher Scientific, Pittsburg, PA) in 6.3 mg/mL penicillin and 10.1 mg/mL streptomycin antibiotic (both from Sigma-Aldrich, St. Louis, MO) RPMI-1640 media. Twenty-four hours after plating, LNCaP cells were treated separately with different SPS (250 nM–1000 nM) with and without testosterone (10 nM) or DHT (1 nM) for 72 hours. The fatty acid and phytosterol concentrations of the 3 SPS used for* in vitro* studies are shown in Table 2. The SPS concentrations used were selected to avoid LNCaP cell cytotoxicity. Both androgens were dissolved in absolute ethanol and the final ethanol concentration in media was 0.1%. These androgen concentrations maximally stimulate LNCaP cell proliferation [29, 30]. SPS stock solution (GNC Herbal Plus SPS (HLLP), Jarrow Formulas SPS (HLHP), and Doctor's Best SPS (HMLP)) was prepared by dissolving supplements to a total fatty acid concentration of 1 M in dimethyl sulfoxide (DMSO, Sigma-Aldrich, St. Louis, MO) and serial dilutions were prepared to concentrations of 0.25 M, 0.5 M, and 0.75 M. Fresh SPS dilutions were prepared and stored at 4°C and used for the 72-hour treatment duration of each experiment. SPS treatments were prepared by dissolving SPS stock solutions (0.25 M–1 M) in media to concentrations of 250 nM–1000 nM SPS. SPS with androgen treatments were prepared daily by dissolving respective SPS stock solutions (0.25 M–1 M) with testosterone (10,000 nM) or DHT (1000 nM) (both from Steraloids, Inc., Newport, RI) in media to concentrations of 250 nM–1000 nM SPS with 10 nM testosterone or 1 nM DHT, respectively. In all cell culture treatments, the final DMSO concentration in media was 0.0001%. Negative controls were treated with DMSO in media (0.0001% v/v). Positive controls for SPS with androgen treatments were treated with 10 nM testosterone or 1 nM DHT and DMSO in media (0.1% v/v for androgens and 0.0001% v/v for DMSO).

The fatty acid and phytosterol molar concentrations of SPS were calculated as follows:

 Concentration = (Quantity of fatty acid/phytosterol in SPS * *(mg/g) × Weight of SPS * *(g))/Volume of SPS* * (mL),
 Molar concentration = (Concentration of fatty acid/phytosterol (mg/mL))/(Molecular weight of fatty acid/phytosterol* * (mg/mmol)).

### 2.4. Cell Number and Cytotoxicity Assays

LNCaP cell number and cytotoxicity were quantified using the CellTiter 96 AQueous One Solution Assay and Cytotox 96 Nonradioactive Cytotoxicity Assay, respectively (both from Promega Corporation, Madison, WI) with a BioTek Synergy HT Plate Reader (BioTek, Winooski, VT) at 490 nm. Cytotoxicity was quantified by measuring lactate dehydrogenase (LDH) released into cell culture media from damaged cells, following SPS treatment with and without androgens. Cell cytotoxicity was calculated as experimental LDH release of the treatment group divided by control and expressed as mean percentage. Three replicates of experiments were completed.

### 2.5. Syrian Hamsters

Five- to six-week-old, castrated male Syrian hamsters were purchased (Harlan Laboratories, Inc., Indianapolis, IN) and acclimated for one week before treatment was initiated. Hamsters were housed individually in plastic cages, with free access to Purina LabDiet 5001 (LabDiet, St. Louis, MO) and water, and maintained on a 12-hour light/12-hour dark cycle. The day before treatment began, the lower back of each hamster was shaved with electric clippers to expose flank organs, a procedure that was repeated weekly during the 21-day study. Hamsters were randomized to control (*n* = 4), HLLP, HLHP, and HMLP SPS (*n* = 6) groups (Table 1). Testosterone or DHT (0.5 *μ*g/day) dissolved in 5 *μ*L of ethanol was applied daily to the right hind flank organ using a pipette and disposable tips. These androgen concentrations increased androgen-sensitive flank organ growth moderately to approximately 15–20 mm^2^ previously, which is 50–70% maximal stimulation [31]. The left hind flank organ served as the control and was treated with ethanol only. Thirty minutes after androgen or ethanol treatment, SPS or ethanol (5 *μ*L) was applied to each flank organ in treatment and control groups, respectively, using a pipette and disposable tips [32]. Flank organ area was calculated weekly by taking 2 diameter measurements 90 degrees apart with an electronic, digital, high-precision Mitutoyo caliper (Tokyo, Japan) and using the formula for area of an ellipse: area = *π∗*(length/2)*∗*(width/2), as previously described [33]. Hamsters were euthanized by CO_2_-induced asphyxiation.

### 2.6. Statistical Analysis

Data were analyzed using SAS 9.3 (SAS Institute Inc., Cary, NC) with *p* < 0.05 considered statistically significant. LNCaP cell number and cytotoxicity results were analyzed using ANOVA with Dunnett's test. For animal studies, paired *t*-test was used to analyze the left and right flank organ areas, and Wilcoxon nonparametric one-way ANOVA was used to analyze the difference between left and right flank organ growth between controls and SPS treatment groups.

## 3. Results

### 3.1. Saw Palmetto Supplements' Fatty Acid and Phytosterol Quantities

Both HLLP and HMLP SPS had relatively high total fatty acids quantities compared to HLHP SPS (Table 2). Total phytosterols quantities in HLHP SPS were 50-fold and ~20-fold higher than in HLLP and HMLP SPS, respectively. The quantities of laurate and myristate were higher in HMLP SPS compared to HLLP and HLHP SPS. The quantity of oleate was high in all three SPS, with the highest quantity observed in HLLP SPS. Linoleate, campesterol, stigmasterol, and *β*-sitosterol quantities were higher in HLHP SPS compared to HLLP and HMLP SPS.

### 3.2. Effect of Saw Palmetto Supplements with and without Testosterone or DHT Treatment on LNCaP Cell Number

**Figure 1:**
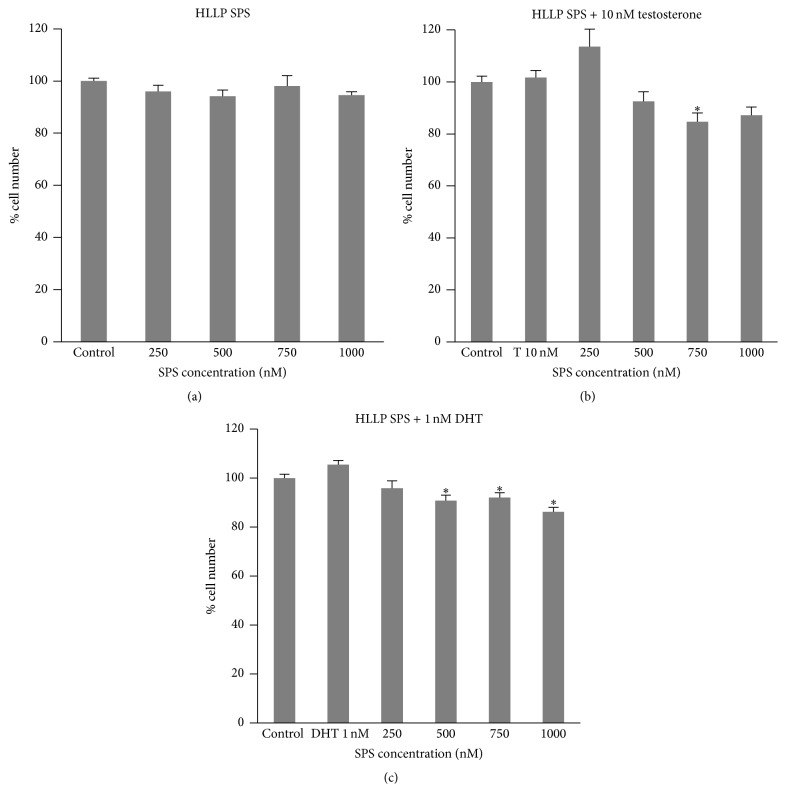
LNCaP cell number after treatment with HLLP SPS with and without 10 nM testosterone (T) or 1 nM DHT for 72 hours. (a) No androgen. (b) Testosterone. (c) DHT. Data obtained from three replicates of each experiment and expressed as mean percentage (± SEM) relative to 0.0001% DMSO control (*n* = 3; ^*∗*^ *p* < 0.05 versus control).

**Figure 2:**
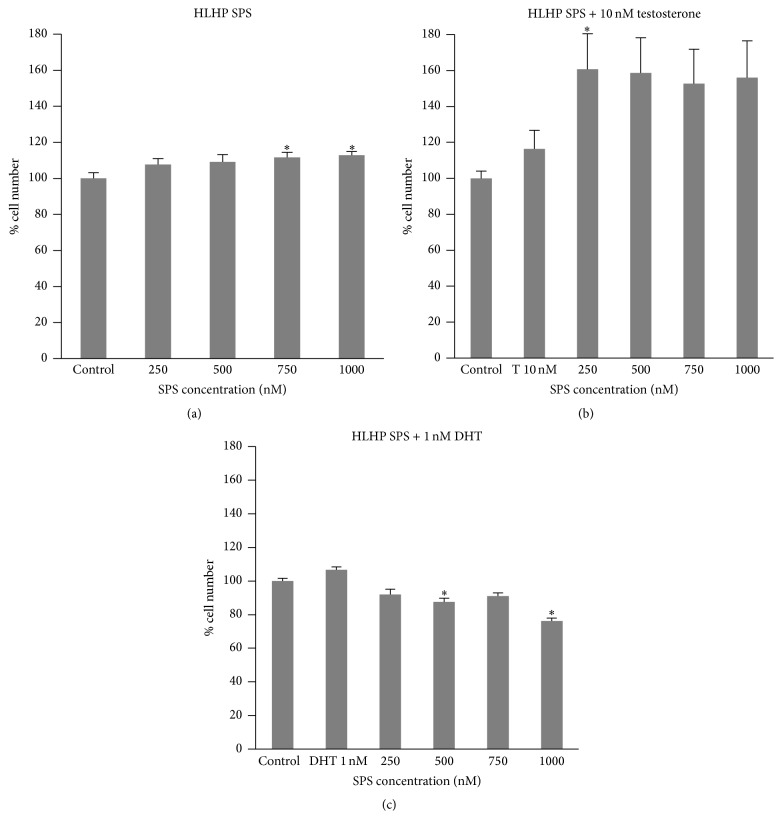
LNCaP cell number after treatment with HLHP SPS with and without 10 nM testosterone (T) or 1 nM DHT for 72 hours. (a) No androgen. (b) Testosterone. (c) DHT. Data obtained from three replicates of each experiment and expressed as mean percentage (± SEM) relative to 0.0001% DMSO control (*n* = 3; ^*∗*^ *p* < 0.05 versus control).

**Figure 3:**
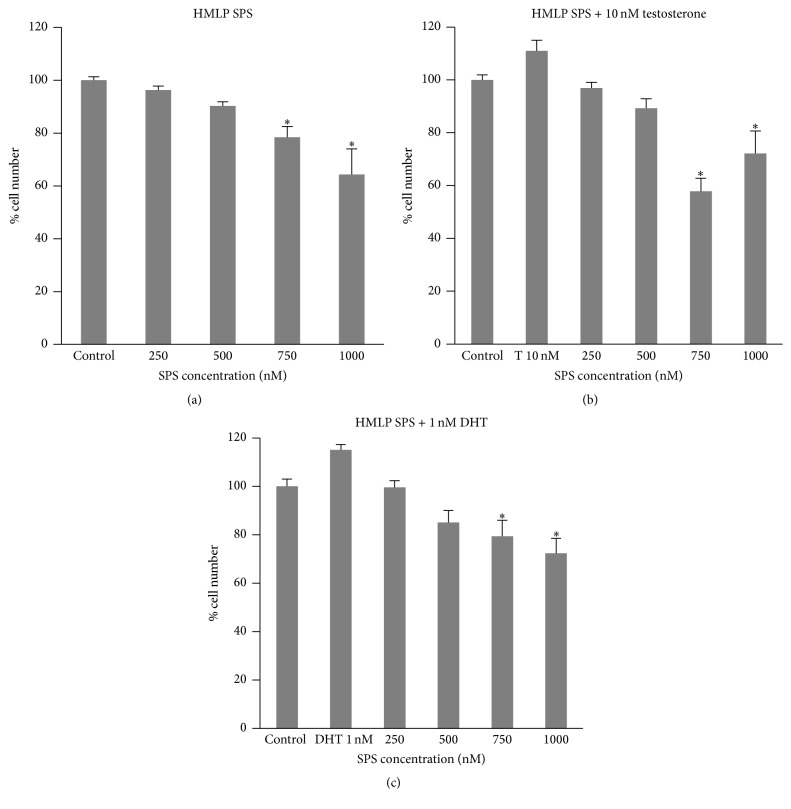
LNCaP cell number after treatment with HMLP SPS with and without 10 nM testosterone (T) or 1 nM DHT for 72 hours. (a) No androgen. (b) Testosterone. (c) DHT. Data obtained from three replicates of each experiment and expressed as mean percentage (± SEM) relative to 0.0001% DMSO control (*n* = 3; ^*∗*^ *p* < 0.05 versus control).

There was no significant increase in LNCaP cell number with testosterone or DHT treatment compared to the control (Figures 1(b), 1(c), 2(b), 2(c), 3(b), and 3(c)). HLLP SPS greater than or equal to 750 nM with testosterone (Figure 1(b)) and greater than or equal to 500 nM with DHT (Figure 1(c)) treatment significantly decreased LNCaP cell number to 85% and 86–92% of the control, respectively. Seven hundred and fifty (750) nM and 1000 nM HLHP SPS treatment without androgens significantly increased LNCaP cell number to 112% and 113% of the control, respectively (Figure 2(a)). Two hundred and fifty (250) nM HLHP SPS significantly increased LNCaP cell number to 160% of the control in testosterone-treated LNCaP cells (Figure 2(b)), and 500 nM and 1000 nM HLHP SPS significantly decreased LNCaP cell number to 88% and 76% of the control, respectively, in DHT-treated LNCaP cells (Figure 2(c)). Seven hundred and fifty (750) nM and 1000 nM HMLP SPS with and without testosterone or DHT treatment significantly decreased LNCaP cell number compared to the control (Figures 3(a), 3(b), and 3(c)). Overall, HMLP SPS at high concentrations inhibited the growth of LNCaP cells compared to HLLP and HLHP SPS; therefore a cytotoxicity assay was performed to determine whether this growth inhibition was due to the toxic effect of HMLP SPS at their respective concentrations with and without androgen treatment. Results showed that HMLP SPS was not cytotoxic to LNCaP cells with and without androgen treatment (Table 3).

### 3.3. Final Body Weights, Food Intake, Flank Organ Areas, and Growth

**Figure 4:**
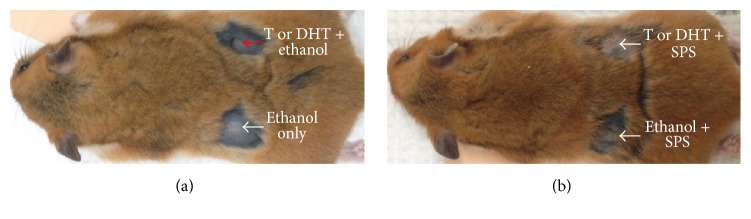
Androgen stimulation and the effect of SPS on androgen-dependent flank organ pigmentation. Right flank organs for controls in both testosterone- (T-) and DHT-treated SPS groups were highly pigmented, as shown with red arrow; the left flank organs were not pigmented, as shown with white arrow (a). No pigmentation was seen in either of the flank organs in the treatment groups, as shown with white arrows (b).

There were no significant differences in final body weights and daily food intake between SPS treatment groups and the control. There were also no significant differences between the left and right flank organ areas in controls and SPS treatment groups (Table 4). However, SPS treatments caused a notable but nonsignificant reduction in the difference between the left and right flank organ growth in the testosterone-treated SPS groups compared to the control. The same level of inhibition was not seen in the DHT-treated SPS groups (Table 5). It should be noted that the right flank organs for controls in both testosterone- and DHT-treated SPS groups were highly pigmented; the left flank organs were not. No pigmentation was seen in either of the flank organs in the SPS treatment groups (Figure 4).

## 4. Discussion

In LNCaP cells, HLLP SPS significantly decreased cell number at high concentrations with testosterone or DHT treatment. HLHP SPS on the other hand increased LNCaP cell number with and without testosterone treatment but significantly decreased cell number at high concentrations with DHT treatment. HMLP SPS significantly decreased LNCaP cell number at high concentrations with and without testosterone or DHT treatment. The antiandrogenic action of SPS has been attributed to their ability to block the conversion of testosterone to DHT by inhibiting 5*α*-reductase or prevent the binding of DHT to androgen receptors [34, 35]. In our study, SPS reduced LNCaP cell number more effectively in the presence of androgens than without them. This result is consistent with the greater inhibition of LNCaP cell growth with saw palmetto berry extract (SPBE) and DHT compared to SPBE alone [36].

It is also important to note that there was no significant difference in LNCaP cell number between testosterone and DHT positive controls and the DMSO control in all SPS treatment groups. Given that fetal bovine serum used in media preparation lacked androgen (personal communication with company), we expected there would be a significant increase in cell number between the androgen-treated LNCaP cells and the control. Previously, LNCaP cell growth was inhibited with 10 nM or 500 nM testosterone added to 10% fetal bovine serum supplemented media [37]. The proposed mechanism for this androgen-mediated growth inhibition is that high DHT prevents stabilization of androgen receptor during mitosis, thus inhibiting cell growth [38–40].

Another possibility is that testosterone and DHT are metabolized so rapidly [41–44] that they are ineffective in stimulating LNCaP cell growth. Synthetic androgens, which have a similar affinity for the androgen receptor as testosterone or DHT and are not metabolized (e.g., methyltrienolone and mibolerone) [33, 45], can be used for* in vitro* studies to stimulate growth of LNCaP cells. It is important to note that these synthetic androgens would not be useful in a study where 5*α*-reductase inhibition is a suspected mechanism, because they will not be acted on and converted to a more potent androgen like testosterone. We performed some studies with 10 nM DHT, but this concentration was not as effective as 1 nM DHT in stimulating LNCaP cell growth, which is consistent with LNCaP cells grown in charcoal-stripped media [29].

In Syrian hamsters, SPS treatments did not significantly reduce the difference between the left and right flank organ growth in testosterone- and DHT-treated SPS groups; however, it caused a notable reduction in the difference in the testosterone-treated SPS groups. The same level of inhibition was not observed in the DHT-treated SPS groups. It is possible that these differences would have been significant if we had larger group sizes. Our group sizes were based on Liang and Liao that reported a greater hamster flank organ growth of 24.9 mm^2^ and 32.1 mm^2^ for testosterone and DHT-stimulated treatment groups, respectively [17], compared to 20.2 mm^2^ and 22.3 mm^2^ for testosterone and DHT-stimulated treatment groups, respectively, in our study. It is possible that part of the reason we did not see as great of a response to treatment was that the flank organ growth was less responsive to androgen treatment. The right flank organs for controls in both testosterone- and DHT-treated SPS groups were highly pigmented, an observation seen previously [17], indicating that the androgens were stimulating flank organ growth and causing pigmentation in the hair shaft and near the orifice of the hair follicles [31]. The lack of pigmentation of flank organs in the treatment groups may indicate that SPS were to some extent inhibiting, or neutralizing, testosterone and DHT stimulation of the androgen-responsive sebaceous glands, dermal melanocytes, and hair follicles [26, 27], all of which contribute to flank organ pigmentation. Alternatively, SPS may interfere with cellular mechanisms in flank organs responsible for the response to androgenic hormones.

In general, the lack of difference in the efficacy of SPS with different nutrient profiles could mean that laurate, myristate, oleate, linoleate, campesterol, stigmasterol, and *β*-sitosterol are not the only bioactive components, or there is a synergistic effect of specific or all fatty acids and/or phytosterols in SPS responsible for their antiandrogenic activity.

## 5. Conclusions

Overall, we did not find much difference in the efficacy of the SPS with different nutrient profiles in inhibiting androgen-sensitive LNCaP human prostate cancer cells and impacting androgen-sensitive Syrian hamster flank organ growth. Further studies are required to clarify our findings and determine if SPS with different nutrient profiles have differing antiandrogenic efficacy.

## Figures and Tables

**Table 1:** Study design.

+ Testosterone (0.5 *μ*g/day)	+ DHT (0.5 *μ*g/day)
Control (ethanol only)	Control (ethanol only)
GNC Herbal Plus SPS (HLLP)	GNC Herbal Plus SPS (HLLP)
Jarrow Formulas SPS (HLHP)	Jarrow Formulas SPS (HLHP)
Doctor's Best SPS (HMLP)	Doctor's Best SPS (HMLP)

**Table 2:** Saw palmetto supplements' (SPS) fatty acid and phytosterol quantities (mg/g) and LNCaP cell culture SPS treatment concentrations based on 1000 nM total fatty acids.

	HLLP SPS	HLHP SPS	HMLP SPS
	Fatty acid quantities (concentration)		
Laurate (C12:0)	83.3 (90.7)	107.2 (133.6)	274.9 (298.8)
Myristate (C14:0)	31.8 (34.6)	42.5 (53.0)	102.9 (111.8)
Palmitate (C16:0)	97.7 (106.3)	85.7 (106.8)	80.7 (87.7)
Stearate (C18:0)	25.5 (27.8)	32.3 (40.2)	18.0 (19.6)
Oleate (C18:1)	551.8 (600.6)	224.6 (279.8)	296.5 (322.3)
Linoleate (C18:2)	68.9 (75.0)	259.1 (322.8)	48.6 (52.8)
Other fatty acids	59.7 (65.0)	51.2 (63.8)	98.4 (107.0)
Total fatty acids	918.7 (1000)	802.6 (1000)	920.0 (1000)

	Phytosterol quantities (concentration)		
Campesterol	0.2 (0.05)	21.5 (5.57)	0.7 (0.16)
Stigmasterol	0.1 (0.02)	10.1 (2.62)	0.3 (0.07)
*β*-Sitosterol	1.0 (0.23)	33.5 (8.68)	2.3 (0.52)
Total phytosterols	1.3 (0.29)	65.1 (16.86)	3.3 (0.75)

**Table 3:** Relative media LDH levels following HMLP SPS treatment on LNCaP cells expressed as mean percentage relative to 0.0001% DMSO control^1^.

Treatment group	Control (%)	T control (%)	DHT control (%)	750 nM (%)	1000 nM (%)
HMLP SPS	100.0 ± 6.5	—	—	95.6 ± 2.2	104.4 ± 4.7
HMLP SPS + T	100.0 ± 9.6	104.4 ± 8.5	—	107.0 ± 8.1	106.2 ± 8.3
HMLP SPS + DHT	100.0 ± 6.6	—	99.7 ± 6.6	97.4 ± 6.6	100.0 ± 7.4

^1^Data are expressed as mean percentage ± SEM (*p* < 0.05).

T: 10 nM testosterone; DHT: 1 nM dihydrotestosterone; —: not applicable; LDH: lactate dehydrogenase.

**Table 4:** Final body weights, daily food intake, and flank organ area in testosterone- and DHT-treated SPS groups^1^.

Treatment of right flank organ with testosterone or DHT + ethanol or SPS	Final body weights (g)	Daily food intake (g)	Flank organ area (mm^2^)	
			Left (untreated)	Right (treated)
Testosterone + ethanol (control)	104.0 ± 2.4	8.7 ± 0.3	19.8 ± 0.7	22.7 ± 2.9
Testosterone + HLLP SPS	110.7 ± 3.5	8.1 ± 0.2	19.2 ± 1.6	18.9 ± 1.3
Testosterone + HLHP SPS	106.4 ± 4.4	8.1 ± 0.2	18.0 ± 0.9	20.2 ± 1.3
Testosterone + HMLP SPS	109.9 ± 4.8	8.3 ± 0.2	17.6 ± 1.4	19.1 ± 1.2
DHT + ethanol (control)	103.6 ± 2.5	7.9 ± 0.2	22.4 ± 1.2	23.5 ± 1.6
DHT + HLLP SPS	103.9 ± 4.3	8.1 ± 0.3	19.0 ± 2.2	22.3 ± 2.4
DHT + HLHP SPS	100.9 ± 5.8	8.4 ± 0.3	18.0 ± 1.4	19.1 ± 1.5
DHT + HMLP SPS	108.4 ± 5.2	7.9 ± 0.2	20.4 ± 1.1	21.9 ± 1.9

^1^0.5 *µ*g testosterone or DHT was dissolved in 5 *µ*L ethanol. Data are expressed as mean ± SEM (*p* < 0.05).

**Table 5:** Difference between left and right flank organ growth in testosterone- and DHT-treated SPS groups^1^.

Treatment of right flank organ with testosterone or DHT + ethanol or SPS	Flank organ growth (mm^2^)		Difference between left and right flank organ growth (mm^2^)
	Left (untreated)	Right (treated)	
Testosterone + ethanol (control)	1.4 ± 2.7	8.5 ± 1.8	7.1 ± 2.3
Testosterone + HLLP SPS	3.2 ± 1.5	2.1 ± 1.2	−1.2 ± 1.9
Testosterone + HLHP SPS	2.2 ± 1.7	2.8 ± 2.6	0.6 ± 1.5
Testosterone + HMLP SPS	1.8 ± 1.5	3.2 ± 1.8	1.4 ± 2.7
DHT + ethanol (control)	0.2 ± 1.7	4.8 ± 3.2	4.6 ± 4.1
DHT + HLLP SPS	5.2 ± 2.2	7.4 ± 1.8	2.1 ± 1.3
DHT + HLHP SPS	0.7 ± 2.0	4.1 ± 2.0	3.4 ± 2.9
DHT + HMLP SPS	4.6 ± 1.9	8.3 ± 3.4	3.7 ± 2.4

^1^0.5 *µ*g testosterone or DHT was dissolved in 5 *µ*L ethanol. Data are expressed as mean ± SEM (*p* < 0.05).
